# Topical bromfenac as adjunctive treatment with intravitreal ranibizumab for diabetic macular edema

**DOI:** 10.1007/s10792-023-02722-1

**Published:** 2023-04-21

**Authors:** Ahmed F. Gabr, Marian F. Kamel, Ahmed A. Elbarawy

**Affiliations:** Ophthalmology Department, Faculty of Medicine, Aswan University, Aswan, Egypt

**Keywords:** Diabetic macular edema, Ranibizumab, Bromfenac, Macular thickness, Intravitreal injection

## Abstract

**Purpose:**

To determine the safety and efficacy of adding topical bromfenac 0.09% in the treatment of diabetic macular edema.

**Methods:**

Seventy patients (70 eyes) with center involved diabetic macular edema with macular thickness (300–500 μm) were included. Patients were divided randomly into two groups: 35 eyes in each group. Both groups were treated with intravitreal ranibizumab monthly for three consecutive months. Bromfenac 0.09% eye drops twice daily was added to the treatment of study group for six months from commencement of treatment. The efficacy of topical bromfenac was evaluated by comparing both groups through follow-up period as regards to visual acuity, central and average thickness and the need for re-injection.

**Results:**

Patients treated with topical bromfenac in addition to intravitreal ranibizumab revealed significant improvement in visual acuity, more reduction in central and average macular thickness and less tendency to need reinjection compared to those treated with ranibizumab alone (*p* 0.013, *p* 0.010 and *p* 0.022, respectively). No side effects was encountered with the use of topical bromfenac.

**Conclusion:**

Topical bromfenac 0.09% twice a day could enhance and sustain the efficacy of intravitreal ranibizumab in the treatment of diabetic macular edema without increasing the incidence of corneal side effects.

## Introduction

Diabetic retinopathy and consequently diabetic macular edema (DME) remains the leading cause of vision loss particularly among older patients with type 2 diabetes [1]. Being multifactorial, the pathogenesis of DME is not yet fully known with hypoxic state stimulates vascular endothelial growth factor (VEGF), causing more edema [2, 3].

Intravitreal injection of anti-VEGF agents has revolutionized the treatment of DME. Ranibizumab represents a monoclonal antibody antigen-binding fragment, which is administered intravitreally to neutralize all isoforms of vascular endothelial growth factor A [4]. But its consecutive monthly injections affect patient’s productivity, cost and time, in addition to the risk of endophthalmitis [5]. Hence, the need for adjuvant therapies is raised to enhance the effects of intravitreal anti-VEGFs or decrease the frequency of injections.

Inflammatory mediators may participate in the progression of vascular disease in patients with diabetic retinopathy. Therefore, nonsteroidal anti-inflammatory drugs (NSAIDs) were suggested to be beneficial in the reduction of vascular permeability and accordingly in the management of diabetic macular edema. Moreover, topical NSAIDs have fewer side effects [6, 7].

Bromfenac is an NSAID with a topical formulation. Its mechanism of action is binding to and inhibiting the activity of cyclooxygenase II (COX II), precluding conversion of arachidonic acid to cyclic endoperoxides, which are precursors of prostaglandins (PG). Its specific differences in the pharmacodynamics and tissue penetration are the reason for its superiority over other agents in its class, in addition to its capability to penetrate into the posterior segment [8].

Considering the high prevalence of DME and its challenging treatment, the present study was conducted to investigate whether addition of topical bromfenac to the intravitreal ranibizumab would promote its efficacy compared with intravitreal ranibizumab only.

## Aim of the work

To determine the efficacy and safety of topical bromfenac 0.09% in addition to intravitreal ranibizumab for the treatment of diabetic macular edema.

## Patients and methods

This prospective randomized clinical trial was performed from May 2021 to March 2022 at Aswan University Hospital. Informed consent was signed by all patients and approval for the study was obtained from the local Institutional Review Board (IRB). Also, the study was in line with the Declaration of Helsinki.

### Sample size

The sample size was carried out using G*Power 3 software. A calculated minimum sample of 60 patients with diabetic macular edema was needed. However, to avoid missed or lost to follow-up patients, we enrolled 70 patients (divided into two groups, each of them including 35 patients). Group (I) received ranibizumab intravitreal injection, and group (II) received ranibizumab intravitreal injection plus additional topical bromfenac 0.09% twice daily for six months.

### Inclusion criteria

Patients aged of 20–60 years old, with evidence of non-proliferative diabetic retinopathy, and centers involving macular edema (average macular thickness of 300–500 μm) who agreed to use additional topical treatments to the standard intravitreal injection of anti-VEGF were included.

### Exclusion criteria

Patients with proliferative diabetic retinopathy, history of cataract surgery in less than one year, intravitreal injection or macular photocoagulation in less than six months and/or any other concomitant ocular pathologies causing vision loss were excluded from the study.

### Methods

After obtaining complete medical and ocular history, all included patients were subjected to full ophthalmological examination including unaided and best-corrected visual acuity, slit-lamp examination for anterior segment, intraocular pressure (IOP) measuring using applanation tonometry and fundus examination using slit-lamp biomicroscopy using Volk 78D fundus lens. Fluorescein angiography was done, and optical coherence tomography (OCT) was performed using (Topcon 3D-OCT-2000 (FA Plus), Tokyo, Japan). Central macular thickness (CMT) represented thickness at the point of intersection of the six radial scans of the OCT, while average macular thickness (AMT) denoted thickness in the central 1000 μm diameter area of the macula.

All the included patients received three consecutive monthly injections of intravitreal ranibizumab *0.05 mL of 6 mg/mL solution* (Lucentis™, Genentech, South San Francisco, CA). Patients were randomly distributed using the lottery method. They received either topical bromfenac 0.09% (Bromoflam™, Orchidia pharmaceutical, Al-Obour City, Egypt) in the first group or artificial tears twice a day over the study period (6 months) in the second group.

The patients were followed up every 4 weeks. At each visit, patients were undergone unaided and best-corrected visual acuity (BCVA) measured using LogMar, slit-lamp examination for anterior segment, IOP measuring using applanation tonometry and fundus examination. Central and average macular thickness were measured by OCT and reported at first, third and sixth months. Each patient had followed up chart divided into squares numbered by month days to ensure patient compliance with eye drops twice daily. Success was measured by improvement in BCVA, decrease signs of non-proliferative diabetic retinopathy (NPDR), decrease macular thickness in OCT, decrease number of intravitreal injections or increase duration of reinjection.

The number of intravitreal injections (IVR) was compared between the study groups and reinjection was administered based on the “as-needed” protocol if average macular thickness was 300 μm or more and visual acuity was not improved. Complications of injections as well as side effects of topical treatment during follow-up period were monitored and reported.

### Statistical analysis

Data were verified, coded and analyzed using IBM SPSS 24.0 (IBM SPSS Inc., Chicago, IL, USA). Descriptive statistics: means, standard deviations, median, range and percentages were calculated. Chi-square/Fisher’s exact test was used to test significance. For continuous variables, independent t-test analysis and two-way repeated measure ANOVA (RM-ANOVA) tests were used appropriately. The clinical and demographic factors with proven statistical significance from the univariate analyses were further included in multivariable logistic regression models. A significant *p* value was considered when it is less than 0.05.

## Results

A total number of 70 eyes from 70 patients who completed the study in two arms (35 eyes in each group) were included for final analysis. The baseline characteristics of the study groups regarding age, gender, duration of diabetes and glycosylated hemoglobin percentage (HbA1C) are demonstrated in Table 1 revealing no statistical differences between both study groups.

**Table 1:** Baseline characteristics of the studied groups

		Ranibizumab group (*n* = 35)	Ranibizumab + bromfenac group (*n* = 35)	*P*-value
Age (years)	Mean ± SD	59.91 ± 6.3	59.11 ± 6.2	0.594*
	Median (Range)	59 (48–72)	59 (48–71)	
Gender ‘No (%)’	Female	18 (51.4)	16 (45.7)	0.632**
	Male	17 (48.6)	19 (54.3)	
Duration of DM (years)	Mean ± SD	13.23 ± 3.1	13.49 ± 3.9	0.897*
HbA1C	Mean ± SD	7.24 ± 0.3	7.23 ± 0.3	0.853*

^*^T-test, **Chi-square test

As regards to visual acuity, there was no significant difference between both groups at baseline and through follow-up period. Compared to baseline, improvement in visual acuity was noted after sixth months in both groups with statistically significant difference in ranibizumab plus bromfenac group (*p* < 0.001). For the interaction between time and group effect, the improvement in visual acuity was more evident in the study group with statistically significant (*p* = 0.013) (Table 2).

**Table 2:** Effect of adding topical bromfenac with intravitreal ranibizumab for diabetic macular edema on visual acuity, intraocular pressure, central macular thickness and average macular thickness in both study groups

(Mean ± SD)	Ranibizumab group (*n* = 35)	Ranibizumab + bromfenac group (*n* = 35)	*P*-value*
*Visual acuity (LogMAR)*			
Baseline	0.663 ± 0.1	0.693 ± 0.2	0.245
1-month	0.654 ± 0.2	0.684 ± 0.2	0.186
3-months	0.629 ± 0.2	0.643 ± 0.1	0.690
6-months	0.617 ± 0.1	0.586 ± 0.1	0.368
*P*-value**	0.077	< 0.001	0.013***
*Intraocular pressure (mmHg)*			
Baseline	14.57 ± 2.2	14.23 ± 2.3	0.532
1-month	14.40 ± 1.4	14.83 ± 1.4	0.097
3-months	14.91 ± 1.6	14.21 ± 1.3	0.060
6-months	14.23 ± 1.7	13.60 ± 1.6	0.127
*P*-value**	0.261	0.159	0.084***
*Central macular thickness (µm)*			
Baseline	378.71 ± 76.7	401.17 ± 73.1	0.214
1-month	374.57 ± 74.4	370.51 ± 78.2	0.846
3-months	349.71 ± 63.9	332.74 ± 71.2	0.298
6-months	369.83 ± 77.6	315.17 ± 61.3	0.013
*P*-value**	0.014	< 0.001	0.010***
*Average macular thickness (µm)*			
Baseline	376.49 ± 47.1	377.34 ± 53.5	0.934
1-month	372.91 ± 47.6	361.32 ± 42.3	0.288
3-months	356.62 ± 41.9	337.62 ± 35.2	0.044
6-months	351.38 ± 51.1	326.06 ± 42.1	0.027
*P*-value**	0.097	< 0.001	0.060***

^*^T-test, **Chi-square test, *** Two-way repeated measure ANOVA

Although fluctuation of intraocular pressure was found, there was no significant difference between both groups at baseline and follow-ups. For the interaction between time and group effect, the reduction in IOP was slightly more evident in the study group with nonstatistically significant (*p* = 0.084) (Table 2).

Significant reduction of central macular thickness (CMT) was noted in both treated groups at the end of follow-up compared to baseline values. At baseline, first month and third months, there was nonstatistically significant difference between both groups (*p* = 0.214, 0.846 and 0.298, respectively). On the other hand, at sixth month, the CMT was significantly lower in ranibizumab plus bromfenac group (*p *= 0.013). For the interaction between time and group effect, the reduction in CMT was statistically significant in ranibizumab plus bromfenac group (*p* = 0.010) (Table 2).

At baseline and first month, there was nonstatistically significant difference in AMT between both study groups (*p* = 0.934 and 0.288, respectively). On the other hand, at third and sixth months, the AMT was significantly lower in ranibizumab plus bromfenac compared with the other one (*p* = 0.044 and 0.027, respectively). Additionally, there was significant reduction in AMT only in ranibizumab plus bromfenac group at the end of study period (*p* < 0.001). For interaction between time and group effect, the reduction in AMT was more evident in ranibizumab plus bromfenac group with statistically nonsignificant difference (*p* = 0.060) (Table 2).

There were eight eyes from ranibizumab group needed reinjection from the fourth to sixth months compared to only two eyes, which needed reinjection in ranibizumab plus bromfenac group (*p* = 0.022) (Table 3). Self-limited ocular discomfort was reported by two patients (5.7%) in ranibizumab group and in five patients (14.2%) in ranibizumab plus bromfenac group, while subconjunctival hemorrhage was reported by three patients (8.6%) in ranibizumab group compared to two patients (5.7%) in ranibizumab plus bromfenac group with nonstatistical differences. No serious complications as endophthalmitis or corneal ulceration have been encountered in both study groups (Table 3).

**Table 3:** Frequency of reinjection and reported side effects in both study groups

	Ranibizumab group (*n* = 35)	Ranibizumab + bromfenac group (*n* = 35)	*P*-value*
*Frequency of injection*			
4th month	1 (2.9%)	0 (0%)	0.022
5th month	3 (8.6%)	1 (2.9%)	
6th month	5 (14.3%)	1 (2.9%)	
*Complications*			
Subconjunctival hemorrhage	3 (8.6%)	2 (5.7%)	0.754
Pain/burning sensation	2 (5.7%)	5 (14.2%)	

^*^Fisher’s exact test

Table 4 shows the multivariable logistic regression model of the independent effect of adding topical bromfenac as an adjunctive treatment with intravitreal ranibizumab for diabetic macular edema. After adjusting for the basic demographic and clinical data, the final model contained two factors, i.e., CMT and AMT.

**Table 4:** Independent effect of adding bromfenac as an adjunctive treatment with ranibizumab for diabetic macular edema: multivariable logistic regression

Predictor	OR (95% CI)	*P*-value
Age/years	0.977 (0.900–1.060)	0.572
Sex (male)	1.225 (0.427–3.278)	0.687
HbA1C (mmol/L)	0.957 (0.108–8.467)	0.968
DM duration/years	1.048 (0.892–1.231)	0.576
CMT at 6 months	0.993 (0.987–0.999)	0.018
Average thickness at 6 months	0.988 (0.978–0.999)	0.031

It was found that one µm decrease in the central macular thickness was associated with 7% (OR = 0.993, 95% CI: 0.987–0.999) increase in the chance of adding bromfenac, and this was statistically significant *(p* = 0.018). Likewise, one µm decrease in the average macular thickness was associated with 12% (OR = 0.989, 95% CI: 0.978–0.999) increase in the chance of adding bromfenac, and this was statistically significant (*p* = 0.031).

## Discussion

Anti-vascular endothelial growth factor (Anti-VEGF) drugs have resulted in improved visual outcomes, while treating diabetic macular edema. To maintain the initial gains in VA, repeated intravitreal injections are needed with subsequent increase of economic burden as well as possible occurrence of serious complications [9, 10].

Although VEGF upregulation plays an essential role in DME, several studies have demonstrated elevated inflammatory factors in the vitreous cavity of patients with diabetic retinopathy [11]. Inflammatory mediators and prostaglandins, such as interleukins and prostaglandin E2, facilitate leukocyte migration, induce blood–retinal barrier disruption and increase vascular permeability leading to exaggeration of edema [12].

Bromfenac is indicated for the treatment of ocular inflammatory diseases and postoperative inflammation. It is more lipophilic than other NSAIDs, facilitating corneal penetration, increasing duration of action and enhancing COX II inhibitory activity, thereby reducing production of prostaglandins and inflammatory cytokines [13, 14].

Due to the paucity of studies reported about using of topical bromfenac after intravitreal ranibizumab for the treatment of DME, our results will be compared with studies reported about using this combination in the treatment of other causes of macular edema or about using other NSAIDs such as topical nepafenac or ketorolac after intravitreal ranibizumab in treatment of DME.

Both bromfenac and nepafenac may be more beneficial in the treatment of DME compared to ketorolac due to their higher levels of posterior segment penetration [15].

The present randomized clinical trial showed that adding topical bromfenac to intravitreal ranibizumab produces additive effects on DME treatment. Both study groups revealed improvement of visual acuity after sixth month follow-up compared to the baseline. Although statistically not significant, the beneficial effect of visual acuity was more evident in patients who received ranibizumab plus topical bromfenac. This was found also to be correlated with the decrease in CMT and AMT at the sixth months of follow-up.

Nikkhah et al. [16] found that using topical ketorolac 0.5% three times a day after intravitreal bevacizumab in the management of diabetic macular edema, the mean change of visual acuity was greater in bevacizumab plus ketorolac group. Grant also suggested a synergistic effect of topical bromfenac on anti-VEGF treatment in inhibiting choroidal neovascularization (CNV) secondary to age-related macular degeneration (AMD). Patients with topical bromfenac had a significant improvement in visual outcomes [17]. Garcia-Gonzalez et al. [18] suggested that patients with diabetic macular edema treated with topical nepafenac, showed statistically significant improvement in visual acuity.

On the other hand, Shimura et al. [19] did not find any improvement in visual acuity when using combination of ranibizumab plus topical bromfenac in treating macular edema secondary to branch retinal vein occlusion.

In a case series, Pinna et al. [20] applied only topical bromfenac twice a day to treat center involved DME. After 30 days, they found nonsignificant improvement in visual acuity. The same result was also reported by other investigators [5]. Other researchers concluded that visual acuity did not’ improved in patients with neovascular AMD, they could not detect a beneficial effect of adding topical bromfenac (0.09%) twice daily over two months [21].

Both study arms showed macular thickness reduction after sixth month of follow-up compared to the baseline. Furthermore, patients who received ranibizumab plus topical bromfenac demonstrated a more statistically significant reduction of central and average macular thickness compared to patients treated by ranibizumab alone.

Gomi concluded that reduction in CMT in exudative age-related macular degeneration between baseline and month 6 tended to be greater in eyes treated with topical bromfenac. Thus, combined bromfenac may strengthen and extend the effect of an anti-VEGF drug [5]. Also, Warren et al. [22] reported that bromfenac reduced the macular thickness in eyes with chronic pseudophakic cystoid macular edema.

Nikkhah et al. [16] concluded that reduction of mean CMT was significantly greater in bevacizumab plus ketorolac group compared with bevacizumab group in the management of diabetic macular edema.

Garcia-Gonzalez et al. [18] also showed statistically significant reduction of macular thickness when using topical nepafenac alone in the treatment of DME. Also, Pinna et al. [20] also found that the difference between the initial and posttreatment mean macular thickness in DME fell just short of statistical significance (*p* = 0.06) after applying topical bromfenac only. While others found that in eyes with noncentral DME, topical nepafenac 0.1% for one year likely does not have a significant effect on OCT-measured retinal thickness [23].

Intraocular pressure was found to be reduced in the ranibizumab plus bromfenac group (although not significant) compared to group treated with ranibizumab only. This finding could be due to the effect of bromfenac on prostaglandin (PG) production particularly prostaglandin E2 (PGE2), which increases the IOP by local vasodilation and increased permeability of blood–aqueous barrier [24, 25].

In the present study, the need for reinjection was significantly less among patients in ranibizumab plus bromfenac group compared to those treated with ranibizumab alone. Grant also noticed significant decrease in the number of ranibizumab injections after using of topical bromfenac with anti-VEGF for inhibiting CNV secondary to AMD [17]. Moreover, other investigators reported the same advantage of this combination during the treatment of macular edema secondary to branch retinal vein occlusion [19]. On the other hand, Nikkhah et al. [16] found that bevacizumab group and bevacizumab plus ketorolac group were comparable regarding number of intravitreal bevacizumab injections.

Both study groups revealed no serious side effects and adding of topical bromfenac to intravitreal ranibizumab did not alternate this finding. On the contrary, decrease corneal sensation as well as corneal melting were reported after using topical bromfenac. Cumulative application of topical bromfenac may cause a neurolytic keratitis like situation, which may trigger the formation of corneal ulcer [26].

The major limitation of this study was the small sample size and shorter study period. Follow-up for longer period and inclusion of larger number of patients is recommended.

## Conclusion

Topical bromfenac 0.09% twice a day could enhance and sustain the efficacy of intravitreal ranibizumab in the treatment of diabetic macular edema without increasing the incidence of corneal side effects.

## Data Availability

The datasets used and/or analyzed during the current study are available from the corresponding author on reasonable request.
